# New technique in harvesting urinary bladder mucosal graft for panurethral stricture reconstruction: A case report

**DOI:** 10.1016/j.ijscr.2025.110999

**Published:** 2025-01-31

**Authors:** Morad Bani-Hani, Hamza Al-labadi, Fadi Sultan, Heba Habazi, Omar Al-khateeb, Batool Habazi

**Affiliations:** a Department of General Surgery and Urology, Faculty of Medicine, Hashemite University, Zarqa, Jordan; b Prince Hamza Hospital, Amman, Jordan; c Faculty of Medicine, Hashemite University, Zarqa, Jordan; d Department of Biology, Faculty of Science, University of Jordan, Amman, Jordan

**Keywords:** Urethral reconstruction, Pan urethral stricture, Urinary bladder graft, Urethroplasty, Buccal mucosal graft, Graft and urethral substitution

## Abstract

**Introduction and importance:**

Urethral stricture poses a challenge for reconstructive urologists particularly in cases involving panurethral disease, as treatment is vital for both urinary and sexual functions. While buccal mucosa grafts (BMGs) are the gold standard for urethral reconstruction, alternatives like penile skin, lingual mucosa and bladder mucosa are often used, particularly in cases of recurrent strictures or contraindications. Urinary bladder mucosal grafts (UBMGs) are ideal due to their histological properties, but conventional harvesting is invasive. This report introduces a novel, less invasive technique for harvesting UBMGs that is both affordable and accessible, showing promising outcomes.

**Case presentation:**

A 40-year-old male patient presented with a 10-year history of nontraumatic urethral stricture disease. The patient had developed a panurethral stricture due to multiple unsuccessful internal urethrotomies and a failed BMG urethroplasty.

**Clinical discussion:**

Given the patient's complex history and a 17 cm panurethral stricture, a decision was made to harvest a UBMG using a combination of transurethral endoscopic and laparoscopic techniques after obtaining informed consent, minimizing invasiveness.

**Conclusion:**

This case report presents a minimally invasive technique for harvesting UBMG as a substitution for urethral reconstruction to treat complex urethral strictures. The method reduces invasiveness and improves access, offering a potential alternative to BMGs, especially for patients with previous failed repairs.

## Highlights

• Addressing urethral stricture reconstruction challenges, focusing on panurethral disease and its impact on urinary function.
• Evaluating urinary bladder mucosal grafts for recurrent strictures when buccal mucosa grafts are contraindicated.
• Introducing a novel, minimally invasive, cost-effective technique for harvesting urinary bladder mucosal grafts.
• Highlighting the promising outcomes of this new technique and its potential impact on reconstructive urology.

## 1 Introduction

Male urethral stricture disease affected up to 0.6 % of certain populations, with nearly 1.5 million office visits annually and $200 million in costs in the year 2000. It raised yearly healthcare expenses by over $6000 per insured patient (1). Furthermore, panurethral stricture disease imposes a unique challenge for urologists (2). The term “panurethral stricture” refers to a condition where a narrowing of the urethra extends across a large portion, often more than 10 cm, leading to significant obstruction in the urinary tract. This obstruction typically results from repeated infections or previous urinary surgeries. The complexity of surgical intervention for urethral strictures is driven by the invasiveness of the procedure, the anatomical positioning of the graft, and the materials chosen for reconstruction (3,4). The most effective treatment for recurring urethral stricture disease and lengthy primary urethral strictures is substitution urethroplasty (5). Ideal urethral grafts for these procedures should be easy to access, minimally invasive, hairless, thin, and durable to support capillary growth in a moist environment (6). Since the 1990s, BMG has become the most commonly utilized allograft (7). Nevertheless, there is a limited amount of oral mucosa available. In complex, panurethral illnesses, it is necessary to harvest enough graft material to guarantee tubularization to a normal diameter throughout the stricture (8). Moreover, tobacco usage, to some extent, prevents the development of healthy oral mucosa, and sexual infections frequently prevent the development of penile skin (9). There aren't many, if any, answers available for similar situations (10). Skin grafts can supply long tissue segments when the buccal mucosa has been harmed by radiation, tobacco, or oral leukoplakia, but they should be avoided in individuals with lichen sclerosus (11). Since it was first reported in 1947, UBMG was considered the best substitute for urethral reconstruction (12). However, despite research amply demonstrating the advantages of the procedure, it has become outdated due to the invasiveness of harvesting the graft (12).

Theoretically, the epithelium of a UBMG has the idealistic qualities to be an ideal graft because it naturally tolerates a wet environment; the graft allows for the development of new capillaries during the 48-h inoculation process (12); and the tissue is easily pliable and can be easily shaped to any length (13). The anterior urethra is the site of most UBMG, which are mainly done to treat redo-hypospadias (12,13,14). The main disadvantage of graft harvesting the invasiveness of the conventional procedure (14). Interestingly, the usage of UBMG alone causes complications at the distal part of the urethra such as meatal prolapse and stricture which signifies the necessity of using another tissue at the distal part of the urethra other than the urinary bladder mucosa. Two reports of a less invasive technique employing a waterjet and holmium have just surfaced. The ability of holmium lasers to adjust the depth of penetration lowers the danger of problems and allows for extremely accurate tissue ablation, reducing harm to neighboring tissues which is a critical aspect of medical operations (15). Waterjets, which use high-pressure water to cut materials without causing thermal damage, are also suitable for delicate procedures (16). This case represents the successful urethral reconstruction using UBMG harvested through an endoscopic technique in a patient managed at a private hospital. It highlights the feasibility and safety of this method. Additionally, the case report aims to discuss the outcomes of this approach compared to traditional surgical methods for managing panurethral strictures. The work has been reported in line with the SCARE 2023 criteria (17).

## 2 Case presentation

A 40-year-old Middle Eastern male patient with a 10-year history of nontraumatic urethral stricture disease presented for further management of a 17 cm pan-urethral stricture. He was referred by his physician due to worsening urinary symptoms, including severe urinary obstruction and recurrent perineal abscesses, which necessitated the use of a suprapubic catheter over the past year. The patient had previously undergone multiple internal optical urethrotomies and a failed BMG urethroplasty.

**Fig. 1:**
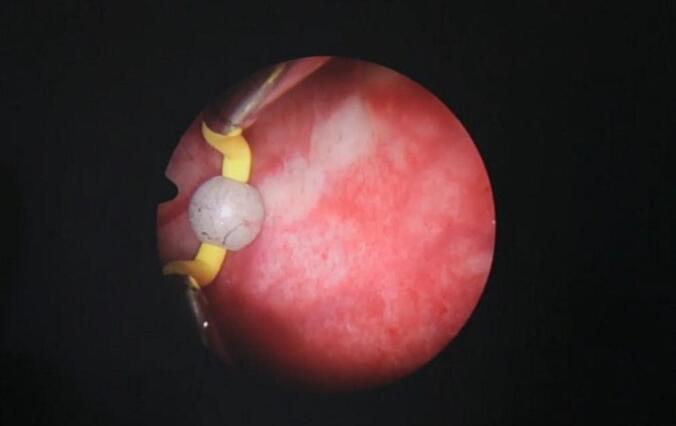
Ball used to mark site of mucosal strip

**Fig. 2:**
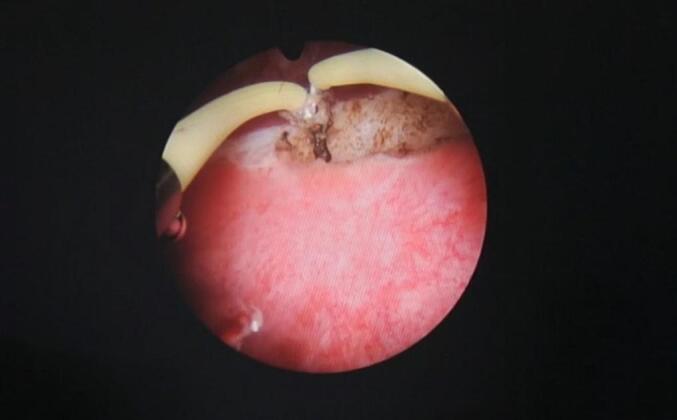
Incision using Collins knife

**Fig. 3:**
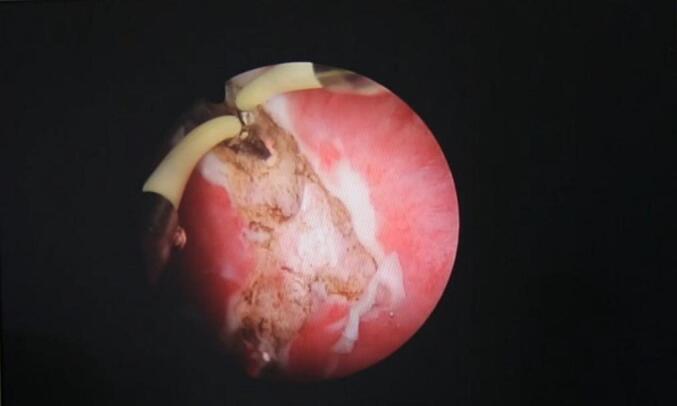
Incision using Collins knife

**Fig. 4:**
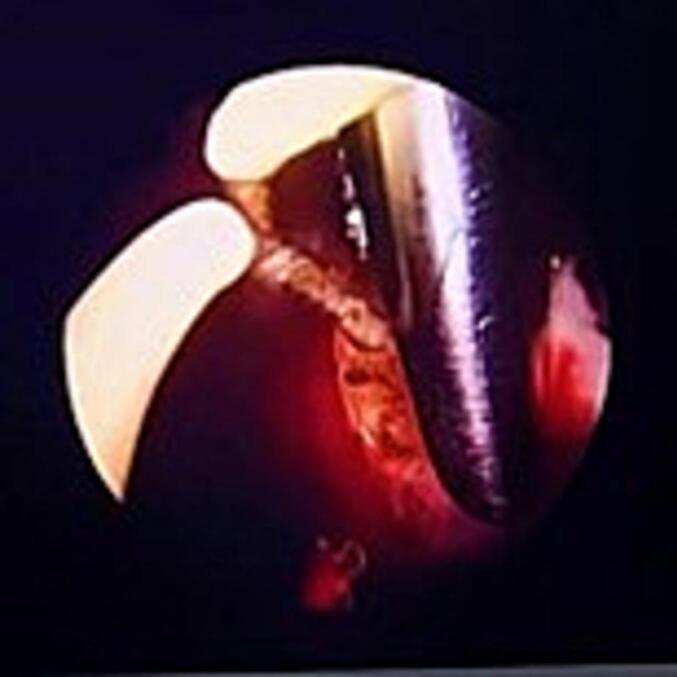
Forceps used to elevate the edge of the mucosal strip

**Fig. 5:**
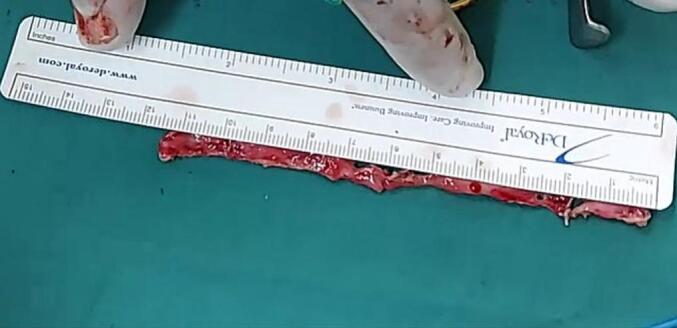
Length of the final graft

**Fig. 6:**
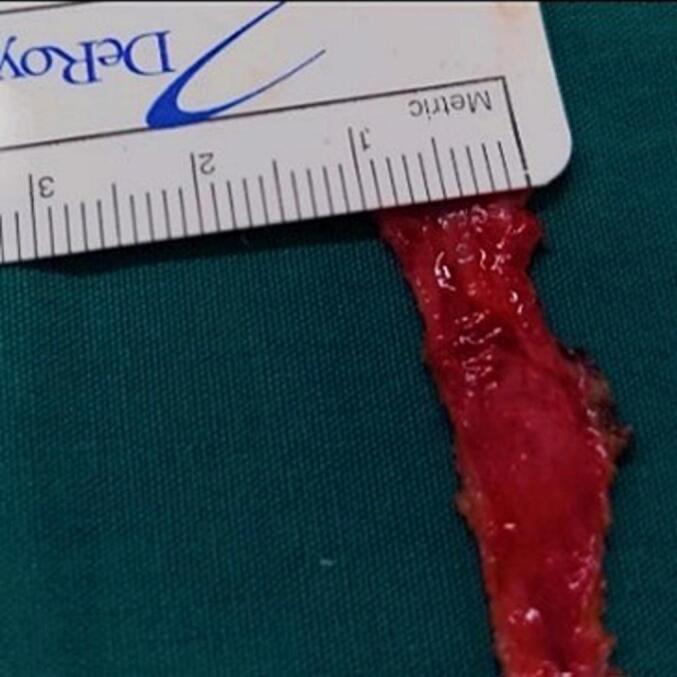
Width of the final graft

After a thorough discussion with the patient and obtaining informed consent, a novel approach to urethral reconstruction was proposed for this patient, which involved a combination of minimally invasive and laparoscopic techniques for harvesting UBMG. The bladder mucosa was harvested using a monopolar resectoscope (set to cutting mode with low power) for transurethral endoscopic resection, ball used to mark site of mucosal strip sparing the trigone (Fig. 1), the incision was made using Collins knife (Fig. 2, Fig. 3). A 5 mm laparoscopic trocar was inserted through the suprapubic catheter tract, and fine grasper forceps were used to assist in elevating edge of mucosal strip (Fig. 4), mucosal strips were extracted through the resectoscope, in this case, because the stricture is long, tissue from two donor sites, the base and the lateral wall is used for the repair. Throughout the procedure the bladder should not be fully distended to avoid inaccurate assessment of the mucosal strip length. Fig. 5, Fig. 6 demonstrate the final view of the graft.

**Fig. 7:**
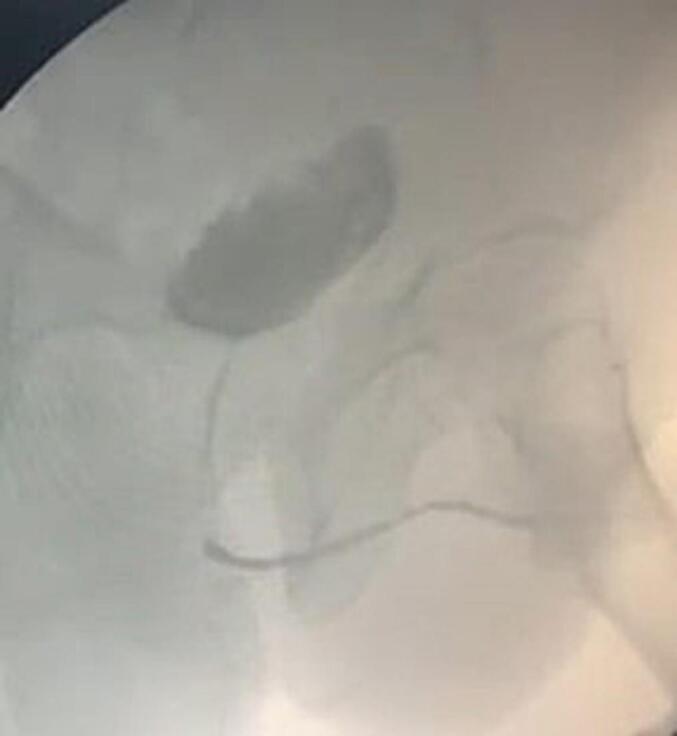
Voiding cystourethrogram

**Fig. 8:**
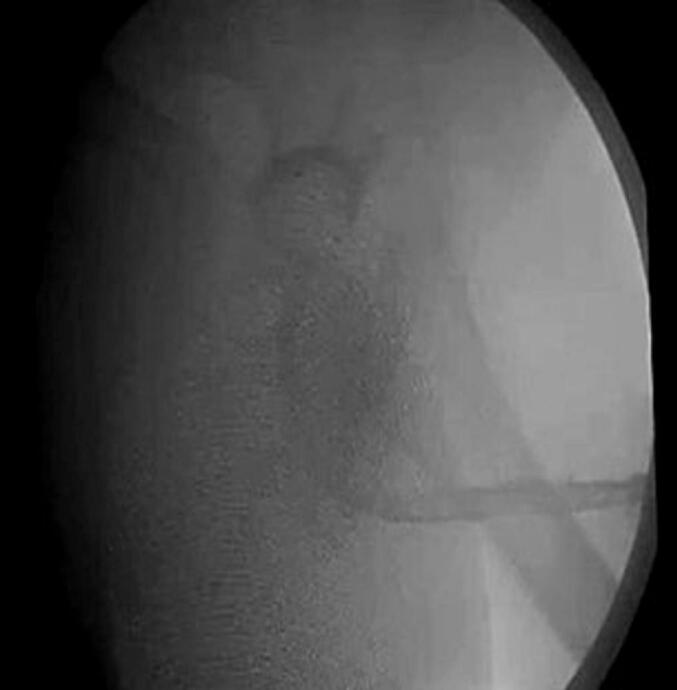
Voiding cystourethrogram

The patient underwent successful urethral reconstruction utilizing UBMG, harvested via endoscopic technique with laparoscopic trocar assistance. No bladder perforation or other postoperative complications were noted. A voiding cystourethrogram (Fig. 7, Fig. 8) in the first case revealed a patent, unobstructed urethra, with no recurrence observed during a six-month follow-up period. However, by the tenth month, the patient developed meatal stenosis, which necessitated further reconstruction using a penile skin flap. To our knowledge, this is the first reported instance of urethral reconstruction employing a UBMG obtained through this particular technique. Transurethral endoscopic surgery may offer a less invasive alternative to traditional open approaches for UBMG harvest. The urethral reconstruction performed with this endoscopic method appears feasible and safe, with promising short-term outcomes.

### 2.1 Procedure details

UBMG was harvested with a resectoscope and laparoscopic trocar, ensuring precision and viability while sparing the bladder trigone.

Fig. 1: ball used to mark site of mucosal strip.

Fig. 2, Fig. 3: Incision using Collins knife.

Fig. 4: Forceps used to elevate the edge of the mucosal strip.

Fig. 5, Fig. 6: final view of graft.

Fig. 7, Fig. 8: Voiding cystourethrogram.

## 3 Discussion

This case demonstrates a novel, minimally invasive technique for treating panurethral stricture disease, a condition that typically presents considerable challenges due to its complexity and the limited solutions for cases where healthy oral mucosa is unavailable, or the quantity of available oral mucosa is limited (18,10). The persistence of the stricture, along with recurrent perineal abscesses and prolonged use of a suprapubic catheter, highlighted the need for an alternative reconstructive method. The perineal abscess was treated with drainage and antibiotics. UBMG, though historically used, was reconsidered in this case due to its idealistic properties, including its natural compatibility with a wet environment due to its epithelium, its flexibility in being tailored to different lengths (13), and the ability to develop new capillaries during inosculation which needs 48 h due to the thinning of the graft (12), making it a suitable option for urethral reconstruction. The surgical team utilized a transurethral endoscopic method supported by a laparoscopic trocar to significantly reduce the invasiveness of the procedure. This approach enabled precise harvesting of the bladder mucosa with the endoscopic resectoscope, while the laparoscopic trocar facilitated manipulation and retraction. Since most patients rely on suprapubic catheters, laparoscopic access was performed in this case through the catheter access port. This minimally invasive approach not only lowered surgical morbidity but also eliminated the need for more invasive open surgery typically associated with harvesting UBMG. The recovery of the patient after surgery showed positive results, with no immediate complications such as bladder perforation. A voiding cystourethrogram performed six months after the procedure confirmed that the reconstructed urethra remained unobstructed. At the ten-month follow-up, the patient developed meatal stenosis, requiring additional reconstruction using a penile skin flap. It is well-established that distal meatal complications are among the most frequent issues associated with UBMG. When exposed to air, the bladder mucosa is prone to metaplastic changes, often becoming adhesive, fragile, and hypertrophic. This process can precipitate meatal stenosis and, in more severe cases, may lead to the formation of a proximal fistula (19).

Advancements such as holmium laser and waterjet-assisted techniques have been explored for harvesting bladder mucosa. By providing extremely accurate tissue ablation with regulated penetration depths, holmium lasers help to minimize harm to nearby tissues and lower the risk of complications during medical procedures. Waterjet cutting is especially appropriate for sensitive procedures since it uses high-pressure water streams to provide precision cutting without thermal damage. While both techniques reduce invasiveness compared to traditional open approaches, they rely on specialized and often expensive equipment, limiting their accessibility in resource-constrained settings (15,16,20).

By contrast, the technique described in this case uses standard laparoscopic and endoscopic tools, providing an affordable and accessible solution, particularly in resource-limited settings. This further underscores its potential for broader applicability.

Urethral reconstruction has long relied on the use of autologous grafts, with BMG being the most widely used graft for complex urethral strictures since the 1990s (7). However, as in this case, BMG is not always effective, particularly in cases of redo surgeries or when the oral cavity, the donor site, is diseased (7,10). UBMG, first reported in 1947, has been largely discontinued due to the invasiveness of the harvesting procedure (12). Most historical reports describe its use in anterior urethral strictures, particularly in redo-hypospadias repairs (12,13,14). The technique described in this case represents a further evolution of minimally invasive methods by combining the monopolar resectoscope with a laparoscopic trocar, offering a more cost-effective and widely applicable solution that could be applied in various healthcare environments. The combination of transurethral endoscopic surgery and laparoscopic assistance via the suprapubic catheter orifice significantly reduces the invasiveness of the procedure, addressing one of the main limitations that has historically prevented the widespread use of UBMG in urethral reconstruction (14).

The successful short-term outcomes observed in this case suggest that UBMG, when harvested using this technique, could be a viable alternative to BMG for patients with complex urethral strictures, particularly in redo cases where other grafts have failed.

Future refinements to prevent complications like meatal stenosis could include optimizing the shape and size of the graft to ensure better integration at the distal urethra and employing adjunctive graft materials, such as buccal or lingual mucosa, for the distal urethral segment.

When oral mucosa isn't an option, penile skin grafts may be considered, though they're less suitable for patients with a history of penile surgery or lichen sclerosus (21). In addition to patients who develop issues from prior hypospadias repairs, such as diverticulum, stricture, fistula, and recurrent chordee (19). Lingual grafts, another alternative when buccal mucosa is limited, share similar qualities to oral tissue but may cause postoperative tongue pain or speech issues (22). These limitations make the UBMG a preferable alternative.

This technique utilizes standard laparoscopic and endoscopic tools rather than specialized equipment, making UBMG harvesting more accessible and potentially affordable. By minimizing invasiveness, it also reduces operating time and associated hospital costs, and fewer complications may decrease readmissions, making it a feasible option across various healthcare settings.

## 4 Limitations

Although the method for harvesting urinary bladder mucosal grafts (UBMGs) that has been described shows promising short-term results, there are a number of drawbacks to take into account. First, since this claim is based on a single case, more extensive research is required to confirm the method's long-term efficacy and applicability across a range of patient demographics. Second, thorough cost evaluations are necessary to evaluate the technique's economic viability, particularly when compared to more sophisticated technology like holmium lasers and waterjet-assisted harvesting or other approaches such buccal mucosal grafts. In order to maximize results and determine which patients will most benefit from this innovative technique, precise patient selection criteria must be created. These limitations highlight the necessity of multicenter trials and further research to fill in these gaps.

## 5 Conclusion

This case report demonstrates the feasibility and potential of a novel, minimally invasive approach for harvesting UBMG to treat complex panurethral strictures. The combination of transurethral endoscopic methods and laparoscopic assistance not only reduces the invasiveness of the procedure but also provides a cost-effective and more accessible solution compared to waterjet and holmium laser techniques. The short-term results are promising, with successful reconstruction and an unobstructed urethra confirmed at the six-month follow-up. While the patient subsequently developed meatal stenosis at the ten-month follow-up, necessitating further reconstruction with a penile skin flap. This report suggests that this innovative UBMG harvesting technique may serve as a viable alternative to BMG in challenging cases of urethral reconstruction, particularly for patients with a history of failed repairs. Further studies are warranted to evaluate long-term outcomes and refine techniques to optimize patient care.

## Author contribution

Conceptualization: Morad Bani-Hani

Acquisition of data: Morad Bani-Hani

Analysis and interpretation of data: Morad Bani-Hani, Hamza Al-labadi, Fadi Sultan, Heba Habazi, Omar Al-khateeb, Batool Habazi.

Methodology: Morad Bani-Hani

Writing of the original draft: Morad Bani-Hani

Writing-review and editing: Morad Bani-Hani, Hamza Al-labadi, Fadi Sultan, Heba Habazi, Omar Al-khateeb, Batool Habazi.

Final approval of the version submitted: Hamza Al-Labadi, Fadi Sultan, Heba Habazi.

## Consent

After a comprehensive discussion of the risks, benefits, and alternatives, the patient provided informed consent to proceed with the proposed novel approach to urethral reconstruction.

## Ethical approval

This case report was conducted in strict compliance with ethical principles, adhering to institutional and international guidelines for ethical research and reporting. Additionally, patient privacy and confidentiality were strictly maintained, with all identifying information anonymized.

## Guarantor

Morad Bani-Hani.

## Funding sources

The authors declare no funding for the research.

## Competing Interests Statement

The authors declare that they have no known competing financial interests or personal relationships that could have appeared to influence the work reported in this paper.
